# Femoral–Obturator–Sciatic (FOS) Nerve Block as an Anesthetic Triad for Arthroscopic ACL Reconstruction: Is This the Magic Trick We Were Missing?

**DOI:** 10.3390/jcm13041054

**Published:** 2024-02-13

**Authors:** Metaxia Bareka, Maria P. Ntalouka, Fragkiskos Angelis, Maria Mermiri, Aikaterini Tsiaka, Michael Hantes, Eleni Arnaoutoglou

**Affiliations:** 1 Department of Anaesthesiology, Faculty of Medicine, School of Health Sciences, University of Thessaly, Larissa University Hospital, 41110 Larissa, Greece; barekametaxia@htomail.com (M.B.); maria.ntalouka@icloud.com (M.P.N.);; 2 Department of Orthopaedic Surgery and Musculoskelatal Trauma, Faculty of Medicine, School of Health Sciences, University of Thessaly, Larissa University Hospital, 41110 Larissa, Greece

**Keywords:** anterior cruciate ligament, obturator nerve block, anesthesia

## Abstract

Arthroscopic anterior cruciate ligament (ACL) reconstruction with hamstring grafting is a common orthopedic procedure that is associated with moderate-to-severe pain. Peripheral nerve blockade as an anesthetic technique is an appealing option in the era of modern anesthesia. The aim of this narrative review is to document the efficacy and safety of the combination of femoral, obturator, and sciatic (FOS) nerve blocks as an exclusive method for anesthesia in patients undergoing ACL reconstruction. An electronic search of the literature published up to October 2023 was conducted in the Medline, Embase, Cochrane, Web of Science, and Google Scholar databases to find studies on ACL reconstruction and peripheral obturator nerve block. Overall, 8 prospective studies—with a total of 315 patients—published between 2007 and 2022 were included in this review. Ultrasound-guided peripheral FOS nerve blockade is an effective anesthetic technique for ACL reconstruction, offering good perioperative pain management, minimal opioid consumption, and an excellent safety profile. Further well-designed prospective studies are needed to determine the best approach for obturator nerve blockade and the appropriate type and dosage of local anesthetic.

## 1. Introduction

Anterior cruciate ligament (ACL) tears are a common knee injury, with more than 100,000 new cases per year in the USA [1,2,3,4,5]. ACL injuries occur mainly in the physically active population, preferentially in women <20 or >40 years of age, and their incidence has increased over the years [5,6,7,8]. Surgical intervention, known as ACL reconstruction, is one of the proposed treatment options [1,2,3,4]. The incidence of ACL reconstruction has increased over the last decades, and ACL reconstruction, which is mainly performed arthroscopically [8], represents one of the most common outpatient orthopedic procedures in ambulatory settings [5,6,7,8].

According to the current literature, “orthopaedic procedures are amongst of the most painful procedure a patient can undergo” [9], resulting in moderate-to-severe postoperative pain. Accordingly, following arthroscopic ACL reconstruction, acute postoperative pain is one of the most common postoperative complications, followed by hemarthrosis [5,10]. Experts suggest that immediate postoperative mobilization is of utmost importance in recovery after ACL reconstruction. However, poor pain control can be a major obstacle to early mobilization [5]. Furthermore, suboptimal postoperative pain control can lead to increased morbidity, delayed recovery, unscheduled emergent hospital admission, and decreased quality of life [5]. Therefore, adequate and appropriate pain management cannot be overemphasized.

General anesthesia, central neuraxial anesthesia (spinal, epidural, combined epidural–spinal), or peripheral nerve block (PNB) are all suitable anesthetic techniques for patients undergoing arthroscopic ACL reconstruction [11]. Performing PNBs before the surgical incision, as part of the concept of pre-emptive multimodal analgesia, leads to improved pain control after surgery, and the success of this treatment has been well documented in joint arthroplasty, resulting in lower pain scores up to 12 months postoperatively [5]. Although multimodal pain management protocols have not yet been established for arthroscopic ACL reconstruction, it appears that the application of PNBs as an anesthetic technique may be the answer we have been missing for optimal postoperative analgesia in these procedures [5,9].

The most commonly described PNBs for arthroscopic ACL reconstruction include the femoral nerve, adductor canal, and sciatic nerve block [5]. However, over the years, several clinical studies have shown that blockade of all three nerves—the femoral, sciatic, and obturator nerves—appears to be essential for adequate pain control after any complex knee surgery, including ACL reconstruction [12,13,14]. The obturator nerve (ON) plays an important role in the innervation of the knee joint capsule [15,16]. Since the anterior branch of the ON innervates the gracilis muscle, ON block should be considered the cornerstone of painless gracilis tendon harvesting [15,16]. However, it seems that there is no clear clinical evidence for the adequacy and effectiveness of three separate PNBs (femoral, obturator, sciatic—FOS) in ACL reconstruction as the sole anesthetic technique. Therefore, our aim is to document the efficacy and safety of the FOS combination as the sole anesthetic method in patients undergoing ACL reconstruction.

## 2. Materials and Methods

### 2.1. Data Sources/Search Strategy

An electronic search of the medical literature published up to October 2023 was conducted using the Medline, Embase, Cochrane, Web of Science, and Google Scholar databases to find studies relevant to ACL reconstruction and peripheral obturator nerve block. Related articles suggested by the PubMed search engine and reviews on this subject were also searched for additional relevant articles. Further articles were also identified via examination of the references cited in the initially identified reports. The search terms were as follows:

- Obturator nerve block;
- Anterior cruciate ligament;
- Anesthesia.

Two reviewers (M.B. and M.P.Nt.) evaluated the eligibility of the studies independently in a non-blinded standardized manner. Disagreements were resolved by discussion with the senior authors (E.A. and M.H.).

### 2.2. Study Inclusion and Exclusion Criteria

To address the main objective, study inclusion and exclusion criteria were structured using the PICOS framework (population, intervention, comparison, outcome, study design).

Population: The population of interest was adult patients undergoing arthroscopic ACL reconstruction under PNBs as the sole anesthetic technique;
Intervention: The investigated intervention was the performance of three separate PNBs, i.e., (i) femoral (F), (ii) obturator (O), and (iii) sciatic (S), abbreviated as the FOS intervention;
Comparison: The comparison was not specified;
Outcome: The effectiveness, the adequacy (measured by incomplete analgesia and failure of the anesthetic technique) and the safety (measured by the appearance of complications) of performing three separate PNBs (femoral, obturator, and sciatic; FOS) as an exclusive method for anesthesia, in patients undergoing ACL reconstruction;
Study selection: All cases, case series, observational or interventional studies (randomized or quasi-randomized clinical trials) were included. Reviews, narrative or systematic, meta-analyses, and qualitative research were excluded. In addition, only articles in the English language were included.

The systematic search strategy based on PICOS criteria is described in Table 1.

### 2.3. Data Extraction

The data extraction and methodological assessment were performed by two independent investigators (M.B. and M.Nt.) until October 2023. The following data were extracted from each study: publication year, country, number of patients, number of study groups, peripheral nerve block technique, dose of local anesthetic for each nerve block performed, percentage of failed sciatic block, rate of conversion to general anesthesia, need for extra analgesic intraoperatively, rate of reported neurological of vascular complications, study of postoperative pain control, and patient’s satisfaction.

## 3. Results

**Figure 1:**
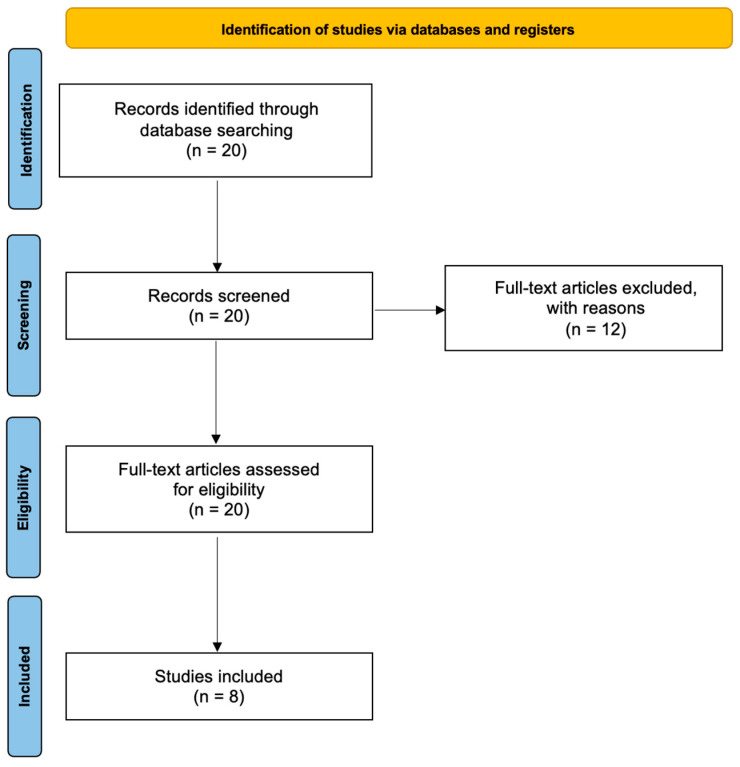
Prisma flowchart.

The initial search identified 20 articles potentially suitable for inclusion (Figure 1). After application of the inclusion criteria, eight articles were retrieved and assessed for eligibility. The final analysis included eight prospective studies, published between 2007 and 2022, in this review [15,16]. The basic characteristics of the included studies are depicted in Table 2.

### 3.1. Number of Patient Groups in Included Studies

Three out of eight studies involved only one group of patients undergoing ACL reconstruction under peripheral nerve blocks, including a separate block of the ON. Four studies included two groups of patients, and one study included three groups of patients, comparing the group of patients undergoing ACL reconstruction under peripheral nerve blockade, including a separate ONB with other peripheral nerve block techniques (Table 2). In total, there were 315 patients receiving FOS nerve block as the anesthetic technique.

### 3.2. Peripheral Nerve Block Technique

In five studies, the FOS nerves were blocked under ultrasound guidance, while in the other three, a more traditional landmark technique was used under neurostimulator guidance. The approach for the ON blockade has shown the biggest heterogeneity (seven different approaches were described).

### 3.3. Local Anesthetic Mixture and Dosage

Based on the results of our study, it seems that there is diversity in both the local anesthetic mixture and the dosage for the peripheral nerve blocks that are being used for ACL reconstruction (Table 3).

### 3.4. Incomplete Analgesia and Failure of Anesthetic Technique

Eleven patients in total had to undergo general anesthesia. The rate of conversion to general anesthesia ranged from 1.72% to 11% (Table 2). At the same time, 36 patients needed intraoperative analgesic supplementation with fentanyl (3–28.5%). In one study, 30% of the patients in the FOS group received intraoperative sedation with propofol at 1 mg/kg/h [17].

**Table 2:** Basic characteristics of the included studies.

Study ID	Type of Study	Number of Patients	PNB Technique	PNB1	PNB2	Evaluation Method for ONB	Incomplete SNB	Conversion to GA	Need for Supplementary Analgesic	Neurovascular Complications	Evaluation of Postoperative Pain Control	Patient or Physician Satisfaction
Helayel [18], 2007 Brazil	Prospective	22	Dual	N/A	FOS	Sensory: Diminished sensitivity or loss of pinprick sensation over the anterior, medial, lateral, and posterior aspects of the thigh and knee Motion: Sphygmomanometer	N/A	No	3 patients (14%)	No	No	N/A
Sakura [14], 2010 Japan	Prospective	21	Dual	F-LFC-S	F-LFC-O-S	Manual evaluation of ON function	1 in 21 (4.8%)	No	6 patients (28.5%)	No	Yes	N/A
Tharwat [19], 2011 Egypt	Prospective	24	NS	PLP-S	FOS	N/A	N/A	2 patients (8.333%)	3 patients (12.5%)	No	Yes	N/A
Taha [17], 2012 United Arab Emirates	Prospective	60	US	N/A	FOS	Hip abduction 40–60°,	3 patients (5%)	No	N/A	No	No	N/A
Simeoforidou [20], 2013 Greece	Prospective	57	Dual	N/A	FOS	Leg elevation. Lifted upwards and laterally, it could not be adducted to the midline.	8 patients (14%)	1 patient (1.75%)	6 patients (10.5%)	No	Yes, morphine 8.6 ± 5.8 mg	
Aissaoui [21], 2013 Morocco	Prospective	20	NS	F-PS	F-O-PS	Adductor strength by sphygmomantometer	0	No	5 patients (25%)	No	No	N/A
Bareka [22], 2018 Greece	Prospective	58	Dual	PLP-S	FOS	Leg elevation. Lifted upwards and laterally, it could not be adducted to the midline.	5 patient (8.6%)	1 patient (1.72%)	11 patients (19.29%)	No	Yes, less morphine consumption than PLP	Patient, refers as good
Goyal [23], 2022 India	Prospective	53	NS	Spinal	FOS	N/A	N/A	7 patients (11%)	2 patients (3%)	No	Yes, less VAS scores and less need for postoperative analgesia	N/A

PNB, peripheral nerve blockade; ONB, obturator nerve block; SNB, sciatic nerve block; GA, general anesthesia; N/A, not applicable; FOS, femoral—obturator—sciatic; F, femoral; LFC, lateral femoral cutaneous; S, sciatic; ON; obturator nerve; PLP, posterior lumbar plexus; NS, neurostimulation, US, ultrasound; PS, parasacral sciatic; VAS, visual analogue scale.

**Table 3:** Type, concentration, and dosage of local anesthetics in each study.

Study ID	LA in FNB	LA in ONB	LA in SNB
Helayel [18], 2007 Brazil	30 mL ropivacaine 0.5%	8 mL ropivacaine 0.5%	15 mL ropivacaine 0.5%
Sakura [14], 2010 Japan	15 mL ropivacaine 0.5%	5 + 5 mL ropivacaine 0.5%	20 mL mepivacaine 1.5% + epinephrine 1:400,000
Tharwat [19], 2011 Egypt	20–30 mL bupivacaine 0.25% + lidocaine 1%	20–30 mL bupivacaine 0.25% + lidocaine 1%	20–30 mL bupivacaine 0.25% + lidocaine 1%
Taha [17], 2012 United Arab Emirates	10 mL ropivacaine 0.33% + 0.67% lidocaine + epinephrine	15 mL ropivacaine 0.33% + 0.67% lidocaine + epinephrine	20 mL ropivacaine 0.33% + 0.67% lidocaine + epinephrine
Simeoforidou [20], 2013 Greece	25 mL ropivacaine 0.5%	10 mL ropivacaine 0.5%	10 mL ropivacaine 0.5%
Aissaoui [21], 2013 Morocco	15 mL bupivacaine 0.25% + lidocaine 1%	6 mL bupivacaine 0.25% + lidocaine 1%	25 mL bupivacaine 0.25% + lidocaine 1%
Bareka [22], 2018 Greece	25 mL ropivacaine 0.5%	10 mL ropivacaine 0.5%	10 mL ropivacaine 0.5%
Goyal [23], 2022 India	15 mL ropivacaine 0.25% + lidocaine 1% + epinephrine	8–10 mL ropivacaine 0.25% + lidocaine 1% + epinephrine	15–20 mL ropivacaine 0.25% + lidocaine 1% + epinephrine

LA, local anesthetic; FNB, femoral nerve block; ONB, obturator nerve block; SNB, sciatic nerve block.

### 3.5. Complications

None of the included studies mentioned any damage to the neurological or vascular structures, or any other complication attributable to the peripheral nerve block. One study [22] referred a 20.6% incidence of shivering in the post-anesthesia care unit (Table 2).

### 3.6. Postoperative Pain Control

Five out of the eight studies present data for postoperative pain (Table 2). Sakura et al. [14] mentioned that two patients in the ON group and one patient from the other group required rescue postoperative analgesia. Simeoforidou et al. stated that the mean total daily dose of morphine postoperatively was 8.6 ± 5.8 mg [20]. Patients with ON block reported higher verbal pain scores postoperatively, accompanied by higher opioid consumption, compared to patients receiving posterior lumbar plexus (PLP) block in Tharwat’s study [19]. Conflicting results came in a later study [22], where the patients in the ON block group consumed less morphine postoperatively compared to the PLP group. Similarly, the study by Goyal et al. [23] reported less postoperative pain and less need for postoperative analgesia in the FOS group.

### 3.7. Patient Satisfaction

Only one study [22] mentioned patient satisfaction. The study reported that all patients were highly satisfied with the perioperative management.

## 4. Discussion

Based on the results of the articles included in this narrative review, we can conclude that arthroscopic ACL reconstruction surgery can be successfully performed under ultrasound-guided peripheral nerve block of the femoral nerve, sciatic nerve, and obturator nerve in an outpatient setting. This approach offers greater patient satisfaction, minimal or no opioid consumption, and an excellent safety profile.

Nowadays, the proliferation of Enhanced Recovery After Surgery (ERAS) protocols, the concept of preemptive multimodal analgesia [9], and the “opioid epidemic” [24] have all contributed to the increased use of regional anesthesia techniques, including PNB. In terms of time management, PNB, when performed in the context of a pre-anesthesia block room, can significantly reduce operating room and post-anesthesia care unit occupancy [25]. In the modern era, more than ever, anesthesia providers need to reduce the contribution of anesthesia practice to global warming, and regional anesthesia appears to be able to reduce greenhouse gas emissions and prevent global warming [26].

To perform ACL reconstruction exclusively under PNBs, the combination of FOS nerve blockade is necessary, as all three nerves contribute significantly to the innervation of the knee. The significance of the ON is more evident when a hamstring graft is used. However, peripheral ON blockade under ultrasound guidance has gained popularity in recent years for a variety of procedures. In addition, several techniques have been described for the use of ON nerve block under ultrasound guidance [15,17,18,20,27,28,29,30,31,32], and some of them have been tested for arthroscopic ACL reconstruction with hamstring autograft [17,18,20]. In 2007, Helayel et al. [18] were the first to describe the performance of ACL reconstruction surgery under obturator, femoral, and sciatic nerve block, and also the first to use dual guidance (ultrasound and neurostimulation), aiming for the common ON. Patients were in the supine position with the hip of the affected side flexed, slightly abducted, and externally rotated, and the knee flexed. A linear ultrasound probe was used. The inguinal ligament and the superior ramus of the pubic bone were used as identification landmarks. The researchers claim that at this location, the ON appears as a fascicular structure accompanied by obturator vessels, located underneath the aponeurotic septum of the pectineus and the adductor longus muscles. The ON was described as a predominantly hyperechoic, flat, or lip-shaped structure in appearance, corresponding to the connective tissue network, with discrete internal hypoechoic dots reflecting the fascicles. The correct placement of the needle was confirmed by neurostimulation. Two other studies [14,17] used an ultrasound interfascial approach to block the ON. Sakura et al. [14] relied on the idea of blocking the anterior and posterior branches of the ON described by Sinha et al. [27]. The obturator nerve was blocked under ultrasound guidance between the adductor longus (or pectineus) and adductor brevis muscles and between the adductor brevis and adductor magnus muscle, respectively. Taha [17] described a proximal interfascial ultrasound-guided technique for blocking the common ON. The patient’s affected limb was abducted and externally rotated. The pectineus muscle was identified by placing a linear probe on the medial aspect of the inguinal crease. By tilting the probe cranially, a hyperechoic structure deep and lateral to the pectineus was visualized. The most medial part of the fascia separating the pectineus muscle from the obturator externus muscle was defined as the injection site. Another dual-guided technique of the common ON was proposed and used by the two remaining studies [20,22]. In these studies, for the ON block, a linear ultrasound probe was positioned opposite from the angle formed by the inguinal crease and the adductor longus. In this position, a sonographic triangle formed by the pectineus, the adductor longus, and the adductor brevis was visible when the probe was tilted appropriately. The investigators described a “spider net” image, next to the pectineus and below the adductor longus, formed by a thick hyperechoic image depicting nerves and connective tissue, and they claim that this reflects the bifurcation of the ON into its two main divisions, the anterior ON and the posterior ON.

Three of the studies included in this review used a neurostimulation-guided technique [19,21,23] based on the techniques described by Macalouet et al. [13], Choquet et al., and Wassef [33], respectively. Of note, in the studies where neurostimulation was used as guidance for the performance of PNB [19,23], the rates of conversion to general anesthesia were higher (8.3% and 11%, respectively). It should be emphasized that there are no studies comparing either the ultrasound-guided techniques to the neurostimulation and landmark techniques or the ultrasound-guided techniques against each other, as far as effectiveness, intraoperative anesthesia, postoperative analgesia, and complications are concerned. Nevertheless, the dominance of ultrasound guidance has revived interest in the ON block in everyday clinical practice, and ultrasound-guided ON block, with or without the simultaneous use of a neurostimulator seems to be the key point for a successful block, regardless of the approach used. Since the ultrasound-guided ON block, the most recent in the FOS combination, demonstrates diversity in the described approaches, evaluating the efficiency of the block has become an appealing goal. The challenge lies in the fact that the sensory block of the ON cannot be consistently and reliably evaluated because the sensory cutaneous distribution of the obturator nerve is highly variable or may even be completely absent. The two studies that have attempted to assess the sensory block of the ON also confirm this [18,19]. For the motor block of the ON, the sphygmomanometer technique by Lang et al. [34] seems to be popular enough [18,21], despite the obvious restrictions that are encountered in the busy theater environment. Simpler techniques that assess the patient’s ability to adduct the hip [17,20] may be more appealing. Nevertheless, if an ON motor block assessment is required, this should be performed and assessed before the induction of the femoral nerve block, which, again, could prove time-consuming.

A notable finding of this review is that the majority of studies report a notable rate of incomplete sciatic nerve block (sensory and/or motor) of up to 14%, but this is consistent with the general failure rate (4–8%) for proximal sciatic nerve blocks in adults [35]. Taking this into account, there is a variable percentage (3–28.5%) of patients who require additional analgesia intraoperatively, usually during graft harvesting. However, the conversion to general anesthesia is low, ranging from 1.72% to 11%, suggesting that the combination of femoral, sciatic, and ON block is an effective approach for peripheral nerve block in ACL reconstruction.

The only alternative feasible choice for performing ACL reconstruction solely under peripheral nerve block is the combination of PLP and sciatic nerve block. There are only two studies comparing these two methods [19,22], and their results are quite contradictory. Tharwat [19] reported that patients under PLP and sciatic nerve block consumed fewer opioids intra- and post-operatively. In contrast, in another study [22], the combination of FOS nerve block resulted in less intraoperative pain and supplemental analgesia, a lower rate of conversion to general anesthesia and significantly less postoperative pain and opioid consumption. A plausible explanation for these contradictory data could be that in the first study [19], all blocks were performed under neurostimulation only, while in the second study [22], all blocks were performed under dual guidance, except for the PLP block, which was performed under neurostimulation. The main disadvantage of the PLP block is that it is a deep block with potentially detrimental complications [36].

The variety of local anesthetics and their concentrations used for PNBs were revealed in this review. Only two studies [19,20] used bupivacaine; all the others used ropivacaine. However, there is great heterogeneity in the concentration of ropivacaine used, ranging from 0.25% to 0.5%, and also in the concomitant use of lidocaine with or without adrenaline as an adjuvant. The basic principle in mind when selecting the appropriate dose and mixture of local anesthetic is to achieve successful anesthesia, prolonged analgesia, rapid mobilization, and avoid adverse outcomes for the patient [37]. The puzzle of the ideal local anesthetic mixture has occupied the scientific community for many years. To date, there are no clear recommendations, and further research is encouraged [38].

Finally, anesthesiologists that perform peripheral nerve blocks are concerned about the rebound pain that appears when the block wears off, which occurs in nearly half of the patients [39]. The two pillars of rebound pain management, as they should also be in postoperative pain management, are patient education and multimodal analgesia [40,41]. The sparse data from the studies providing information on postoperative pain control suggest that the combination of FOS nerve block resulted in good postoperative analgesia with low opioid consumption, and it could be considered a minimal opioid anesthesia and analgesia technique, especially as part of a pre-emptive multimodal regimen, with the appropriate adjustments.

Peripheral nerve blocks as the exclusive anesthetic technique for ACL reconstruction are underused (less than 10% of all anesthesia) [11]. This narrative review is an attempt to promote the FOS combination for anesthesia in ACL reconstruction. The quadruple nerve block (femoral, femoral lateral cutaneous, obturator, and sciatic) that has been used [25,42] may be useful in longer surgical procedures, such as revision ACL reconstruction.

### Limitations of the Study 

This paper is a narrative review, and the results may lack the strengths of a more systematic approach. There are only eight studies providing data for the performance of ACL reconstruction exclusively under three peripheral nerve blocks, including the block of the ON, with a small number of patients. Moreover, the majority of the patients were young (18–49 years old), ASA I–II, and none were obese. Patients with ACL revision surgery, severe bleeding disorders, infection at the sites where the blocks were to be applied, diabetes mellitus or peripheral neuropathy, neurologic deficits to the affected limb, known allergy to the study drugs, body mass index (BMI) >35 kg/m^2^, psychiatric disorders, and communication difficulties were excluded. Therefore, it is not known how these results would apply to older patients with comorbidities. Moreover, there was great heterogeneity in terms of both dosage and local anesthetics used in the studies. Last but not least, none of the included studies investigated the costs or surgeons’ satisfaction, while only one study investigated the patients’ satisfaction.

## 5. Conclusions

In conclusion, it seems that arthroscopic ACL reconstruction surgery with hamstring autograft can be successfully performed under ultrasound-guided peripheral FOS nerve block in an outpatient setting. This approach offers effective perioperative pain management, minimal opioid consumption, an excellent safety profile, and higher patient satisfaction, despite the heterogeneity in the obturator nerve block approach. However, the best dosage and local anesthetic regimen have not yet been determined. Larger studies that include older adults and patients with comorbidities are needed to determine the most reliable technique and identify potential complications.

## Figures and Tables

**Table 1:** Systematic search strategy based on PICO criteria.

**Frame**	**P** **(Patients, participants, population)**	**I** **(Intervention)**	**C** **(Comparator/reference test)**	**O** **(Outcome)**	**Study design**	**Time**	
	Adult patients undergoing arthroscopic ACL reconstruction under PNBs as the sole anesthetic technique	The performance of 3 separate PBNs: (i) the femoral (F); (ii) the obturator (O); (iii) the sciatic (S); abbreviated as the FOS intervention	Not specified	The effectiveness and the adequacy of performing three separate PBNs (femoral, obturator, sciatic; FOS) as an exclusive method for anesthesia in patients undergoing ACL reconstruction	English language	Search period: 1964–October 2023	Last search: (October 2023)
**Inclusion criteria**	Cases, case series, observational or interventional studies (randomized or quasi-randomized clinical trials)						
**Exclusion criteria**	Reviews, narrative or systematic, meta-analyses, and qualitative research						
**Sources**	Databases (Medline, Embase, Cochrane, Web of Science, Google Scholar, and medRxiv) Reference list						

## Data Availability

No new data were created or analyzed in this study. Data sharingis not applicable to this article.
